# Induction of Pluripotent Protective Immunity Following Immunisation with a Chimeric Vaccine against Human Cytomegalovirus

**DOI:** 10.1371/journal.pone.0003256

**Published:** 2008-09-22

**Authors:** Jie Zhong, Michael Rist, Leanne Cooper, Corey Smith, Rajiv Khanna

**Affiliations:** Australian Centre for Vaccine Development, Tumour Immunology Laboratory, Division of Immunology, Queensland Institute of Medical Research, Brisbane, Australia; University of California San Francisco, United States of America

## Abstract

Based on the life-time cost to the health care system, the Institute of Medicine has assigned the highest priority for a vaccine to control human cytomegalovirus (HCMV) disease in transplant patients and new born babies. In spite of numerous attempts successful licensure of a HCMV vaccine formulation remains elusive. Here we have developed a novel chimeric vaccine strategy based on a replication-deficient adenovirus which encodes the extracellular domain of gB protein and multiple HLA class I & II-restricted CTL epitopes from HCMV as a contiguous polypeptide. Immunisation with this chimeric vaccine consistently generated strong HCMV-specific CD8^+^ and CD4^+^ T-cells which co-expressed IFN-γ and TNF-α, while the humoral response induced by this vaccine showed strong virus neutralizing capacity. More importantly, immunization with adenoviral chimeric vaccine also afforded protection against challenge with recombinant vaccinia virus encoding HCMV antigens and this protection was associated with the induction of a pluripotent antigen-specific cellular and antibody response. Furthermore, *in vitro* stimulation with this adenoviral chimeric vaccine rapidly expanded multiple antigen-specific human CD8^+^ and CD4^+^ T-cells from healthy virus carriers. These studies demonstrate that the adenovirus chimeric HCMV vaccine provides an excellent platform for reconstituting protective immunity to prevent HCMV diseases in different clinical settings.

## Introduction

Human cytomegalovirus (HCMV) is a classic example of a group of herpes viruses, which is found universally throughout all geographic locations and socioeconomic groups, and infects 50% of adults in developed countries [1]. Although HCMV does not cause clinical disease in immunocompetent individuals except as a mononucleosis-like illness which is observed in a small number of infected individuals, HCMV infection is important to the following three high-risk groups: 1) unborn babies with an immature immune system, 2) people who work with children, and 3) immunocompromised people such as organ transplant patients and HIV-infected individuals) [1]. Epidemiological studies have shown that 15%–30% of unborn babies who acquire congenital HCMV infection display a variable pattern of pathological sequelae within the first few years of life that may include hearing loss, vision impairment and mental retardation [2]. It has been estimated that in the US alone, each year 8000 newborns have health problems as a results of congenital HCMV infection, with each child costing the US health care system more than $300,000 [3]. Based on the cost and human suffering that would be relieved by reducing the disease burden associated with HCMV infection, the development of a vaccine to prevent HCMV infection or disease was assigned the highest priority, together with vaccines for HIV, TB and Malaria, by the Institute of Medicine (USA) in 1999 [4].

It is now well documented that both humoral and cellular (including CD4^+^ T cells and CD8^+^ T cells) immune responses play an important role in the control of HCMV infection and disease [1], [5]. Therefore a formulation based on viral antigens that activate both humoral and cellular immunity is crucial for a successful HCMV vaccine [6], [7]. During the last 30 years, various strategies, including whole virus, subunit vaccines based on recombinant gB protein, vector vaccines expressing immunodominant antigens (gB protein, pp65 and/or IE-1 protein), DNA vaccine and dense bodies have been developed, and some of these formulations have shown encouraging results in preclinical studies and can even induce HCMV-specific immune responses in some clinical studies [8], [9], [10], [11]. However, none of these vaccines have shown convincing clinical efficacy in the control of HCMV infection or disease, and a clinically licensed HCMV vaccine is still not available.

In recent years, increasing evidence has shown that HCMV-specific immune responses are not restricted to gB, pp65 and IE-1 antigens as previously understood, but are directed towards more than 70% of the HCMV reading frames [12], [13], [14], [15]. Therefore, a vaccine which can induce a broad repertoire of HCMV-specific immune responses in different ethnic populations is likely to provide more effective protection against virus-associated pathogenesis. To achieve this goal, we have designed a novel chimeric vaccine based on a replication deficient adenovirus which encodes 46 HCMV T cell epitopes from 8 different HCMV antigens, restricted through multiple HLA class I and Class II alleles, as a polyepitope [16]. This polyepitope was covalently linked to a truncated form of HCMV-encoded gB antigen which allowed the expression of the HCMV polyepitope and gB proteins as a single fusion protein. Pre-clinical evaluation of this recombinant polyepitope vaccine in HLA A2 transgenic mice (referred to as HHD-2) and humans showed that this formulation is capable of inducing pluripotent cellular and humoral immunity *in vivo* and also readily recalls and expands HCMV-specific CD8^+^ and CD4^+^ T cells.

## Results

### Immunisation of HHD-2 mice with Ad-CMVpoly and/or Ad-gB vaccine induces multiple antigen-specific cellular and humoral immunity

**Figure 1:**
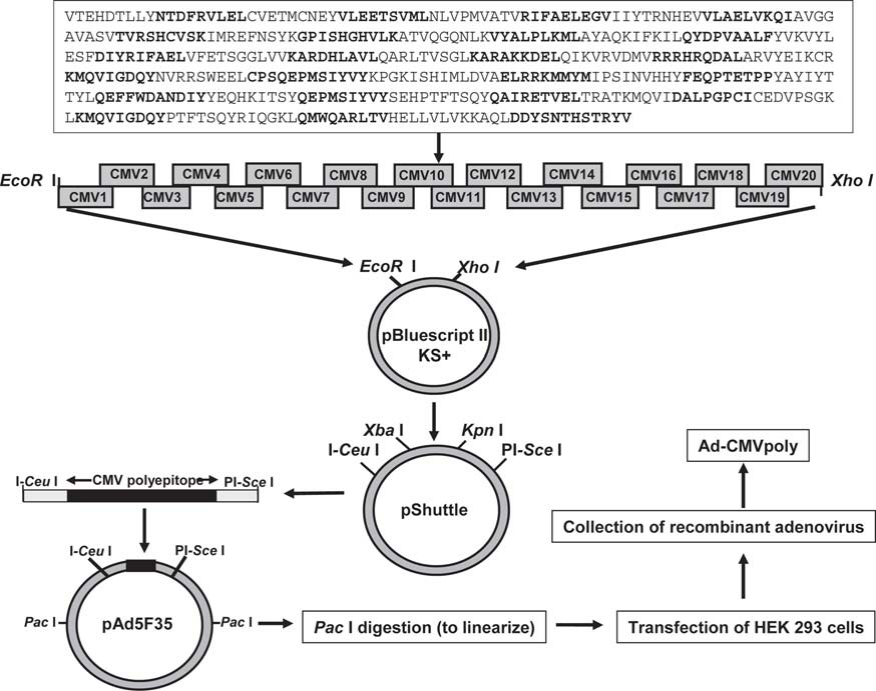
Schematic representation of the construction of a recombinant adenovirus that expresses a synthetic DNA encoding for a polyepitope protein which contains 46 HCMV T-cell epitopes (see box and Table 1). Each of the alternate epitope sequences are shown in bold letters. The DNA sequence encoding this polyepitope protein was constructed using overlapping epitope sequence specific primers (referred to as CMV1 to CMV20) as described in the “Material and Methods” section. This synthetic insert was first cloned into a pBluescript II KS^+^ phagemid, prior to cloning into the pShuttle vector. After amplification in *E.coli*, the expression cassette from pShuttle was excised and ligated into the Ad5F35 expression vector. Following linearization of the DNA using Pac I restriction enzyme, the recombinant Ad5F35 vector was packaged into infectious adenovirus by transfecting HEK 293 cells, and recombinant adenovirus (referred to as Ad-CMVpoly) was harvested from transfected cells by repeated freeze-thawing cycles.

**Figure 2:**
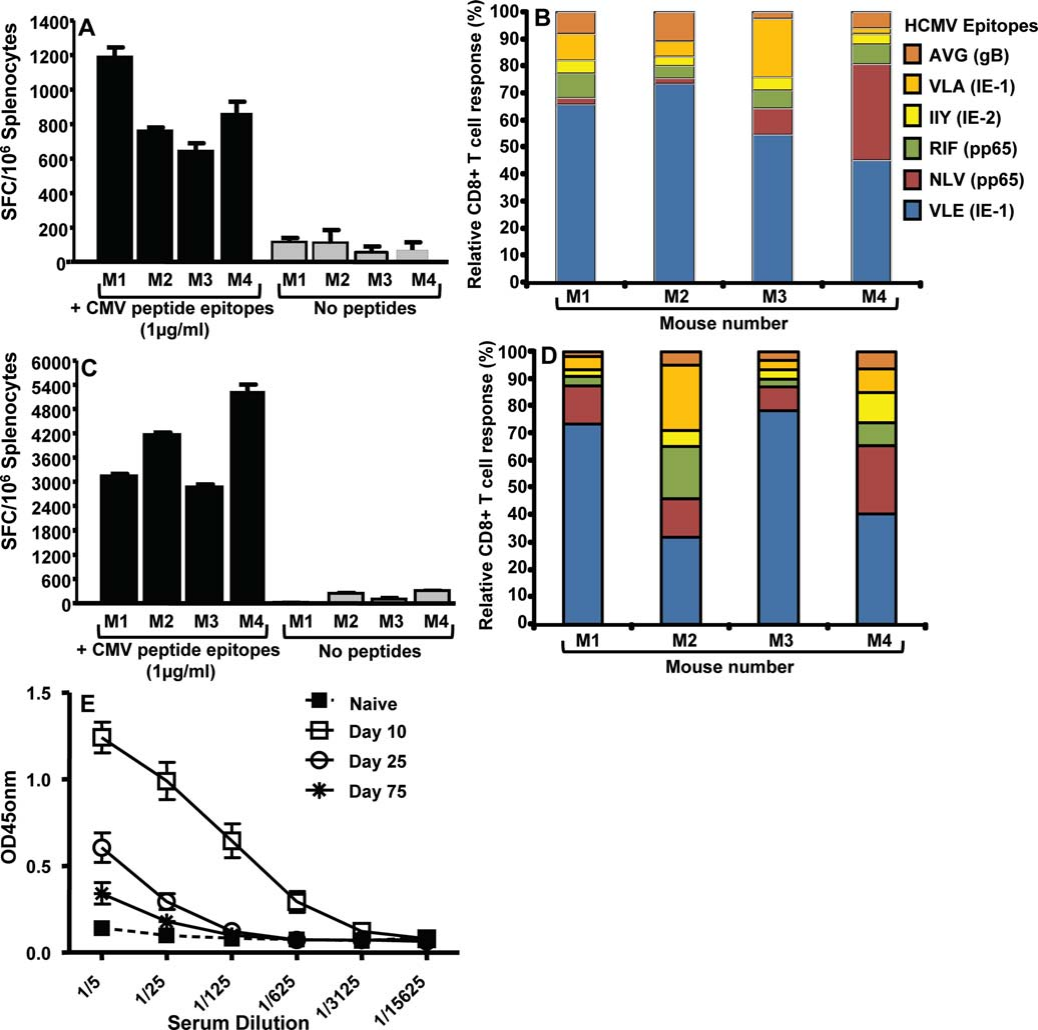
HCMV epitope-specific T cell response following primary and secondary immunisation with Ad-CMVpoly vaccine. Two different groups of HHD-2 mice were immunised intramuscularly with Ad-CMVpoly (7.5×10^8^ PFU/mouse). *A & B*, Following primary immunisation, animals were sacrificed 10 days post immunisation and HCMV epitope-specific reactivity was assessed in the splenocytes by ELISPOT assays as described in the “Material and Methods” section. The epitopes tested for T cell reactivity were VLE (IE-1), NLV (pp65), RIF (pp65), VLA (IE-1), IIY (IE-2) AVG (gB). *C & D*, For immunological analysis following secondary immunisation, animals were given booster immunisation (7.5×10^8^ PFU/mouse) 100 days after primary immunisation and then sacrificed 10 days post secondary immunisation. HCMV epitope-specific reactivity was assessed as described above. *A & C* shows ELISPOT data based on the pooled HLA A2-restricted HCMV epitopes, while *B & D* shows relative T cell responses to individual epitopes. The results are expressed as Mean±SE of spot forming cells (SFC) per 10^6^ splenocytes from four individually tested mice. *E,* Anti-adenovirus antibody titre induced by immunisation with Ad-CMVpoly. Serum samples were collected at different time points after immunisation and anti-adenovirus titres were evaluated by ELISA as described in the “Material and Methods” section. All statistical analyses were conducted using GraphPad Prism 4 software.

Recent studies on the immune regulation of HCMV in healthy virus carriers and transplant patients have clearly indicated that long-term protection from viral pathogenesis is critically dependent on the induction of cellular immunity which is directed towards multiple viral antigens expressed during different stages of HCMV infection [12], [13], [14], [15], [17]. In the first set of experiments we specifically designed our vaccine strategy to induce a cellular immune response against multiple antigens of HCMV using the polyepitope technology [18]. HLA A2 transgenic mice (referred to as HHD-2) were immunised (intramuscularly) with an adenoviral vector encoding 46 HLA class I and II-restricted T cell epitopes as a polyepitope (referred to as Ad-CMVpoly; 7.5×10^8^ pfu/mouse; Figure 1 and Table 1). Ten days after immunisation, *ex vivo* T cell reactivity to the HLA A2-restricted peptide epitopes (pooled; see Table 1) was assessed by ELISPOT technology. It is important to mention here that the virus dosage for vaccination was selected based on our preliminary studies where a range of varying doses were investigated (data not shown). Splenocytes were used as responder cells for the detection of epitope-specific T cells. Data presented in Figure 2A shows that we consistently observed a strong HCMV epitope-specific T cell response following Ad-CMVpoly vaccination. Analysis of T cell responses to the individual epitopes within the polyepitope sequence indicated that during primary immunisation the dominant T cell response was directed towards the VLE epitope derived from IE-1 antigen, although subdominant responses towards other HLA A2-restricted epitopes NLV (pp65), RIF (pp65), VLA (IE-1), IIY (IE-2) AVG (gB) was also detected (Figure 2B).

**Table 1:** List of HLA-restricted HCMV T cell epitopes included in the Ad-CMVpoly and Ad-gBCMVpoly.

Epitope order number	Epitope Sequences	HLA restriction	HCMV Antigens	Parent protein (UL)	Amino acid location	Abbreviated code	Reference
1	VTEHDTLLY	A1	pp50	UL44	245–253	VTE	[13]
2	KPGKISHIMLDVA	B35/DR3	pp65	UL83	283–295	KPG	[13]
3	NTDFRVLEL	A1	gB	UL55	657–665	NTD	[13]
4	VLEETSVML	A2	IE1	UL123	316–324	VLE	[13]
5	NLVPMVATV	A2	pp65	UL83	495–503	NLV	[55]
6	RIFAELEGV	A2	pp65	UL83	522–530	RIF	[13]
7	IIYTRNHEV	A2	IE2	UL122	244–251	IIY	[16]
8	CVETMCNEY	A1	IE1	UL123	279–287	CVE	[13]
9	VLAELVKQI	A2	IE1	UL123	81–89	VLA	[13]
10	AVGGAVASV	A2	gB	UL55	731–739	AVG	[13]
11	TVRSHCVSK	A3	pp50	UL44	52–60	TVR	[13]
12	IMREFNSYK	A3	gB	UL55	682–690	IMR	[13]
13	GPISHGHVLK	A11	pp65	UL83	16–24	GPI	[56], [57]
14	AYAQKIFKIL	A23/A24	pp65?	UL83	248–257	AYA	[13]
15	QYDPVAALF	A24	pp65	UL83	341–349	QYD	[13]
16	YVKVYLESF	A26	pp65	UL83	223–231	YVK	[13]
17	DIYRIFAEL	A26	pp65	UL83	519–527	DIY	[13]
18	VFETSGGLVV	A29	gB	UL55	420–429	VFE	[56], [57]
19	KARDHLAVL	B7	pp150	UL32	101–109	KARD	[13]
20	KARAKKDEL	B7/B8	IE1	UL123	192–200	KARA	[13]
21	TRATKMQVI	B57/B58/Cw6	pp65	UL83	211–219	TRA	[13]
22	HELLVLVKKAQL	DR11	gH	UL75	276–287	HEL	[58]
23	DDYSNTHSTRYV	DR7	gB	UL55	216–227	DDY	[58]
24	QIKVRVDMV	B8	IE1	UL123	88–96	QIK	[13]
25	RRRHRQDAL	B8/B27	pp65	UL83	539–547	RRR	[13]
26	ARVYEIKCR	B27	DNAse	UL98	274–282	ARV	[13]
27	NVRRSWEEL	B7	pp150	UL32	212–220	NVR	[13]
28	CPSQEPMSIYVY	B35	pp65	UL83	103–114	CPS	[16]
29	QARLTVSGL	B7	pp65	UL83	158–166	QAR	[13]
30	ELKRKMMYM	B8	IE1	UL123	199–207	ELK	[13]
31	IPSINVHHY	B35	pp65	UL83	123–131	IPS	[59]
32	FEQPTETPP	B41	IE2	UL122	381–389	FEQ	[16]
33	YAYIYTTYL	B41	gB	UL55	153–161	YAY	[16]
34	QEFFWDANDIY	B44/DRw52	pp65	UL83	511–521	QEF	[13]
35	YEQHKITSY	B44	pp50	UL44	372–380	YEQ	[13]
36	QEPMSIYVY	B44	pp65	UL83	106–114	QEP	[13]
37	SEHPTFTSQY	B44	pp65	UL83	364–373	SEH	[13]
38	QAIRETVEL	B57/B58	pp65	UL83	331–339	QAI	[16]
39	CEDVPSGKL	B40/60	pp65	UL83	232–240	CED	[60]
40	KMQVIGDQY	B40/60	pp65	UL83	215–223	KMQ	[60]
41	ATVQGQNLK	A11	pp65	UL83	501–509	ATV	[60]
42	HERNGFTVL	B40/60	pp65	UL83	267–275	HER	[60]
43	DALPGPCI	B51	pp65	UL83	546–552	DAL	[60]
44	VYALPLKML	A24	pp65	UL83	113–121	VYA	[61]
45	PTFTSQYRIQGKL	B38/DR11	pp65	UL83	367–379	PTF	[55], [62]
46	QMWQARLTV	B52	pp65	UL83	155–163	QMW	[30]

Recent studies have raised some concerns on the use of adenoviral vectors in humans as the pre-existing immunity to adenovirus may compromise the efficacy of these vaccine formulations [19], [20], [21]. To explore this issue, Ad-CMVpoly immunized HHD-2 mice were rested for one or three months and then immunized with the Ad-CMVpoly vaccine (7.5×10^8^ pfu/mouse). Although secondary immunization of mice after one month of vaccination showed very minimal increase in the T cell response (data not shown), a 3–5 fold increase in the T cell response was observed in HHD-2 mice vaccinated after three months of their primary vaccination (Figure 2C). More importantly, following secondary immunization, a small but significant increase in the subdominant responses was observed in some animals, while the T cell response towards VLE epitope remained the most dominant component of overall response (Figure 2D). It was interesting to note that the hierarchy of these T cell responses in HHD-2 mice was very similar to that observed in HLA A2-positive healthy virus carriers [12], [13], [14]. This observation was co-incident with the dramatic decline of antibodies against adenovirus vector 2 to 3 months after immunisation with Ad-CMVpoly (Figure 2E).

### Combination of Ad-CMVpoly and Ad-gB induces long lasting memory cellular and humoral immune responses

**Figure 3:**
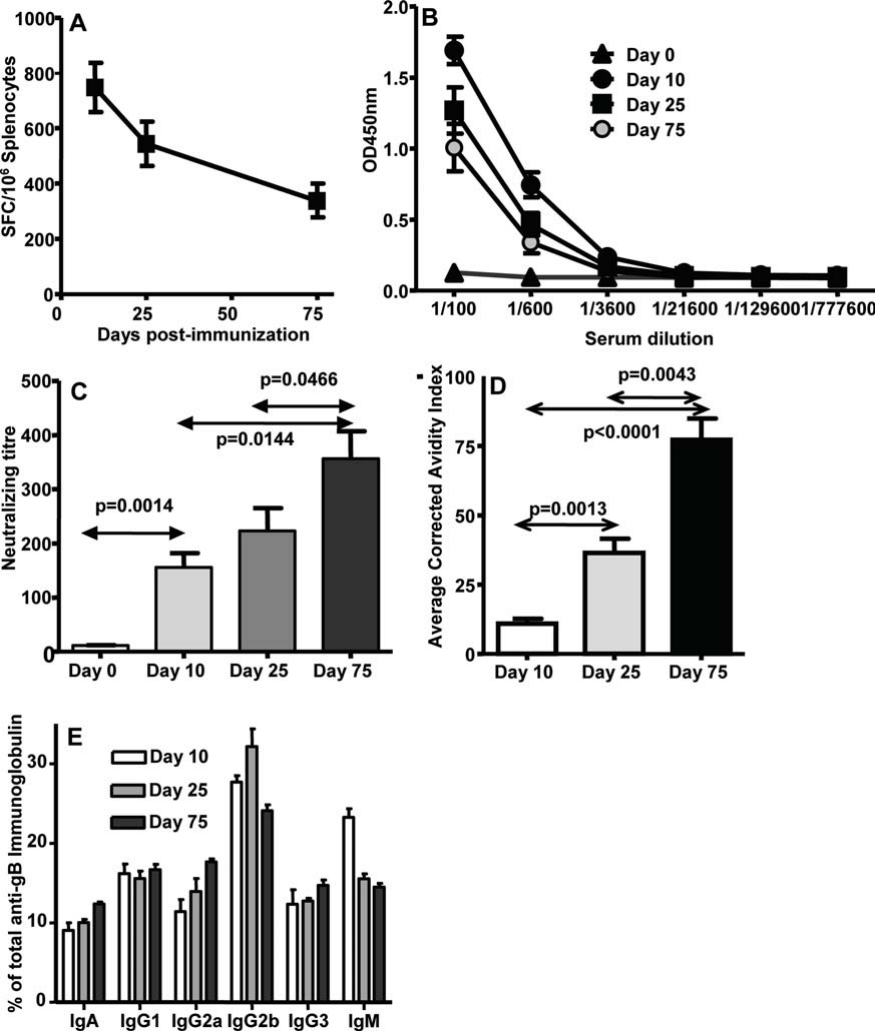
HCMV-specific effector and memory cellular and humoral immune responses following immunisation with a mixture of Ad-CMVpoly and Ad-gB vaccines. *A,* HCMV-specific CD8+ T cell responses following immunisation with Ad-CMVpoly and Ad-gB. These T cell responses were assessed using ELISPOT assays on day 10, 25 and 75 post immunisation. The results are expressed as Mean±SE of spot forming cells (SFC) per 10^6^ splenocytes. *B,* gB-specific antibody responses in serum samples from immunised mice on days 10, 25 and 75. Serum samples on day 0 were collected before the immunisation. *C,* Virus neutralizing capacity of antibody responses induced in HHD-2 mice immunised with Ad-CMVpoly and Ad-gB. Serum samples from these mice were pre-incubated with HCMV virus Ad169 and then these virus preps were used to infect MRC-5. Following overnight incubation virus infectivity was assessed using IE-1/IE-2 expression as outlined in the “Material and Methods” section. *D,* Avidity maturation of gB-specific antibody responses in Ad-CMVpoly and Ad-gB immunised mice. *E,* Immunoglobulin subclass analysis of gB-specific antibody responses in HHD-2 vaccinated mice. Serum samples were collected from three different groups of mice on days 10, 25 and 75 post-immunisation. A minimum of five mice from each group were assessed for HCMV epitope-specific T cell reactivity and humoral immune responses. All statistical analyses were conducted using GraphPad Prism 4 software.

It is now firmly established that although T cell responses play an important role in controlling persistent HCMV infection, humoral immune responses also contribute significantly in controlling primary HCMV infection and as well reduce viral load by neutralizing the extra-cellular virus [22], [23]. To ensure that our vaccine strategy can induce both cellular and humoral immune responses, we immunised HHD-2 mice with the mixture of Ad-gB (7.5×10^8^ pfu/mouse) and Ad-CMVpoly (7.5×10^8^ pfu/mouse), and anti-HCMV specific cellular and humoral immune responses in immunised animals were evaluated at different time point by ELISPOT and ELISA respectively. Data presented in Figure 3A & B shows that this vaccination strategy induced both CD8^+^ T cells and gB-specific antibody responses. The levels of CD8^+^ T cells induced by this co-immunisation strategy were comparable to those seen with Ad-CMVpoly alone and were detectable at reasonably high levels on day 75 post-immunisation (Figure 3A). The levels of gB-specific antibody responses were maintained at high levels by day 75 post-immunisation, although a small reduction was observed when compared to the levels observed on days 10 and 25 respectively. In contrast, the antibody response showed significant increase in the virus neutralization capacity by day 75 post-immunisation (Figure 3C) which was co-incident with the antibody avidity maturation (Figure 3D). It is important to mention here that this increase in neutralization capacity was not due to antibody isotype switching (Figure 3E), which was consist with previous studies [24]. These observations suggested that co-delivery of HCMV polyepitope and gB vaccine can induce long lasting memory T cells immunity and antibody responses.

### Covalent linking of HCMV polyepitope with extracellular gB induces pluripotent T cell and antibody responses

**Figure 4:**
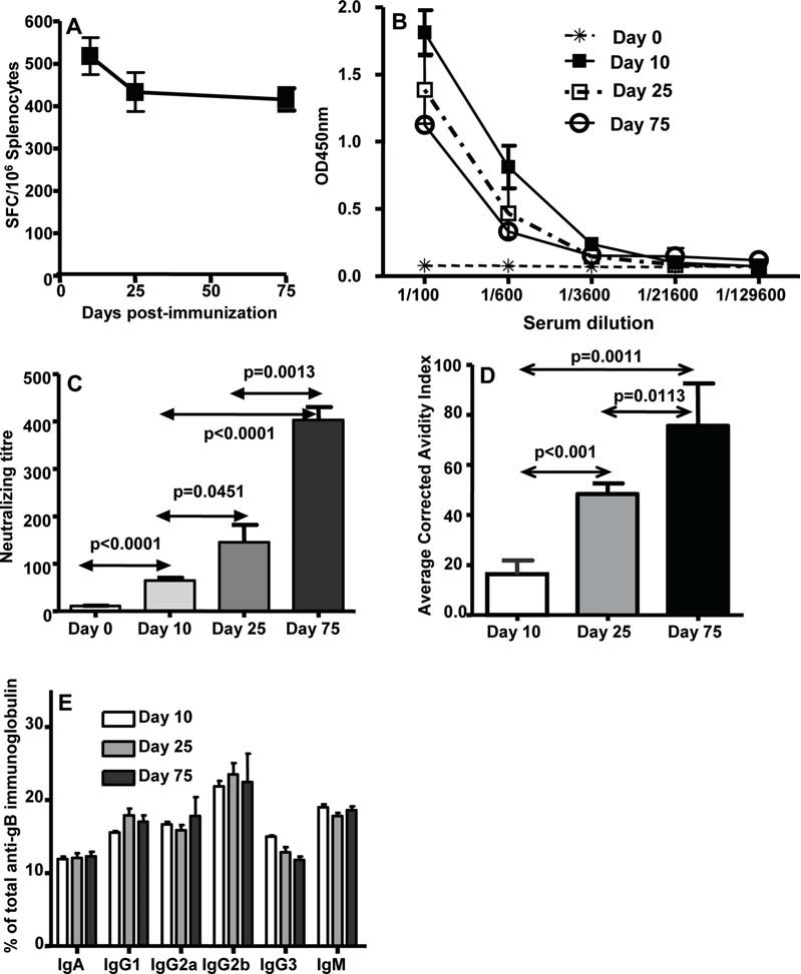
HCMV-specific effector and memory humoral and cellular immune responses following immunisation with Ad-gBCMVpoly vaccine. *A,* HCMV-specific CD8+ T cell responses following immunisation with Ad-gBCMVpoly. These T cell responses were assessed using ELISPOT assays on day 10, 25 and 75 post immunisation. The results are expressed as Mean±SE of spot forming cells (SFC) per 10^6^ splenocytes. *B,* gB-specific antibody responses in serum samples from immunised mice on days 10, 25 and 75. Serum samples on day 0 were collected before the immunisation. *C,* Virus neutralizing capacity of antibody responses induced following immunisation with Ad-gBCMVpoly. Serum samples from these mice were pre-incubated with HCMV virus Ad169 and then these virus preps were used to infect MRC-5. Following overnight incubation virus infectivity was assessed using IE-1/IE-2 expression as outlined in the “Material and Methods” section. *D,* Avidity maturation of gB-specific antibody responses in Ad-gBCMVpoly immunised mice. *E,* Immunoglobulin subclass analysis of gB-specific antibody responses in HHD-2 vaccinated mice. Serum samples were collected from three different groups of mice on days 10, 25 and 75 post-immunisation. A minimum of five mice from each group were assessed for HCMV epitope-specific T cell reactivity and humoral immune responses. All statistical analyses were conducted using GraphPad Prism 4 software.

Although co-immunisation with Ad-gB and Ad-CMVpoly induced both humoral and cellular immune responses against HCMV, delivery of this formulation in a human setting may face significant regulatory constraints. To overcome this potential limitation, we constructed another recombinant adenovirus expressing the extracellular domain of gB and HCMV polyepitope as a single polypeptide (referred as Ad-gBCMVpoly). HHD-2 mice were immunised with the Ad-gBCMVpoly vaccine (7.5×10^8^ pfu/mouse) and both humoral and cellular immune responses were evaluated at the indicated time points. Data presented in Figure 4A shows that immunisation with Ad-gBCMVpoly vaccine induced a long-tem memory CD8^+^ T cell response towards the HLA A2-restricted epitopes from HCMV. Furthermore these animals also showed strong gB-specific antibody response and similar to the data presented in Figure 3B, the levels of gB-specific antibody dropped by day 75 post immunisation (Figure 4B). A significant increase in the neutralizing activity of the antibody response was observed (Figure 4C), which was co-incident with avidity maturation (Figure 4D). On the other hand, there was no antibody isotype switching at different time point after immunisation (Figure 4E). These observations clearly demonstrated that covalent linking of the gB with the polyepitope sequence does not impair the immunogenicity of each of the components of the vaccine.

**Figure 5:**
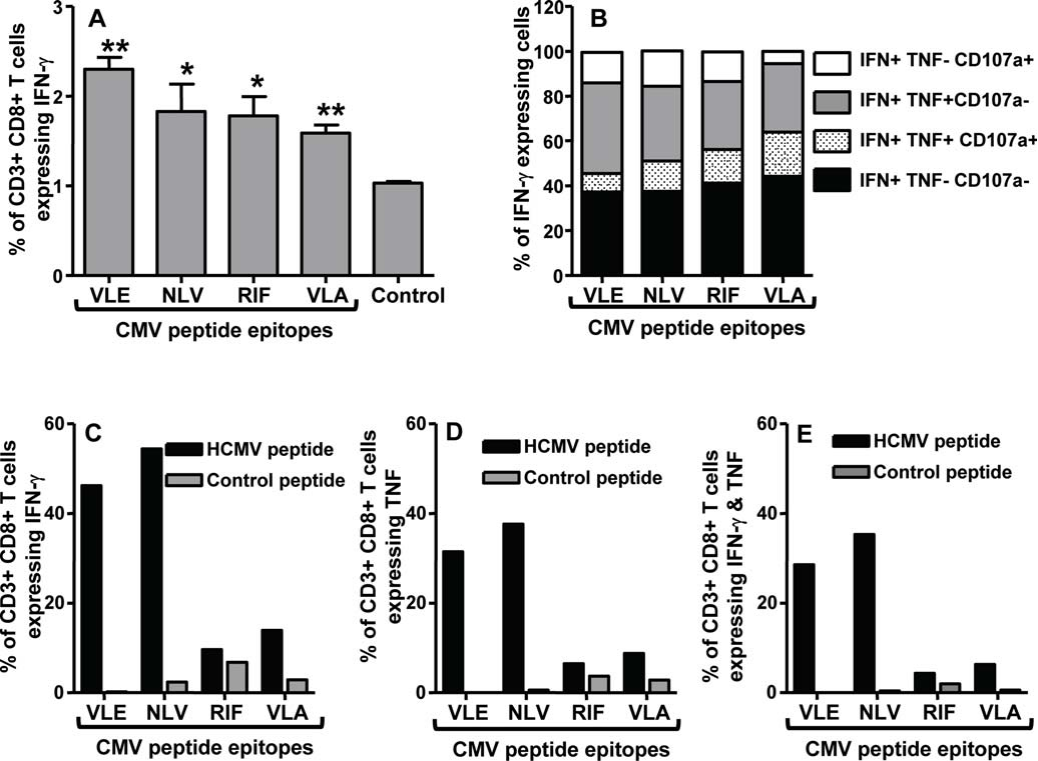
Cytokine expression by HCMV-specific CD8^+^ T cells from Ad-gBCMVpoly immunised HHD-2 mice. *A & B,* Ex vivo expression of IFN-γ, TNF-α and CD107a by antigen-specific CD8^+^ T-cells from Ad-gBCMVPpoly vaccinated mice 10 days post-vaccination. Splenocytes were prepared from 3 individual HHD-2 mice 10 days post-vaccination and cultured with individual HCMV peptides overnight. Anti-CD107a antibody and Brefeldin A was added during the last 6 and 5 hours incubation respectively, followed by T cell surface marker and intracellular cytokine staining. Data represent the percentage of IFN-γ expressing CD8^+^ T cells (*A*) and percentage of single, double or triple markers expressing cells among IFN-γ expressing CD8^+^ T cells (*B*). *C–E,* Expression of IFN-γ and/or TNF-α by *in vitro* expanded antigen-specific CD8^+^ T-cells from Ad-gBCMVPpoly vaccinated mice 10 days post-vaccination. Splenocytes pooled from three immunised mice were first stimulated with individual HCMV peptide epitope-pulsed splenocytes for 2 weeks in the presence of recombinant mouse IL-2 at the concentration of 10 IU/ml, then cultured with MRC-5 cells pulsed with corresponding HCMV peptide epitope overnight for intracellular cytokine assay. The HCMV peptide epitopes tested here were VLE (IE-1), NLV (pp65), RIF (pp65), VLA (IE-1) at the concentration of 1 µg/ml. Data represent the percentage of IFN-γ (*C*), TNF-α (*D*) and IFN-γ & TNF-α (*E*) expressing CD8^+^ T-cells. ** (p<0.005) and * (p<0.05) show statistically significant difference between indicated CMV peptide epitopes and control epitope (*A*). Data from one out three experiments with similar results was shown in *C–E*. All statistical analyses were conducted using GraphPad Prism 4 software.

To further characterize the T cell responses induced by Ad-gBCMVpoly vaccine, we next assessed whether immunisation with Ad-gBCMVpoly result in the differentiation of antigen-specific T cells into fully functional effectors. A number of recent studies have demonstrated that the production of TNF-α in addition to IFN-γ by T-cells is a characteristic of greater differentiation and can enhance protection against infectious pathogens [25], [26], and the translocation of CD107a from intracellular lysosomal and endosomal compartments to the surface of CD8^+^ T cells is a positive marker of degranulation, a requisite process of perforin-granzyme mediated killing function of CTLs [27], [28]. We assessed the level of TNF-α and/or CD107a expression by IFN-γ expressing CD8^+^ T-cells using intracellular cytokine assays. Data presented in Figure 5A–B shows that following *ex vivo* stimulation with HCMV epitopes, CD8^+^ T cells from these mice showed strong IFN-γ expression and a large proportion of these T cells also expressed TNF-α and/or CD107a. Furthermore, after *in vitro* stimulation with individual HCMV peptides, these HCMV peptide-specific CD8^+^ T cells could be expanded and expressed both IFN-γ and TNF-α (Figure 5C–E).

### Protection against quasi-virus challenge following immunisation with Ad-gBCMVpoly

**Figure 6:**
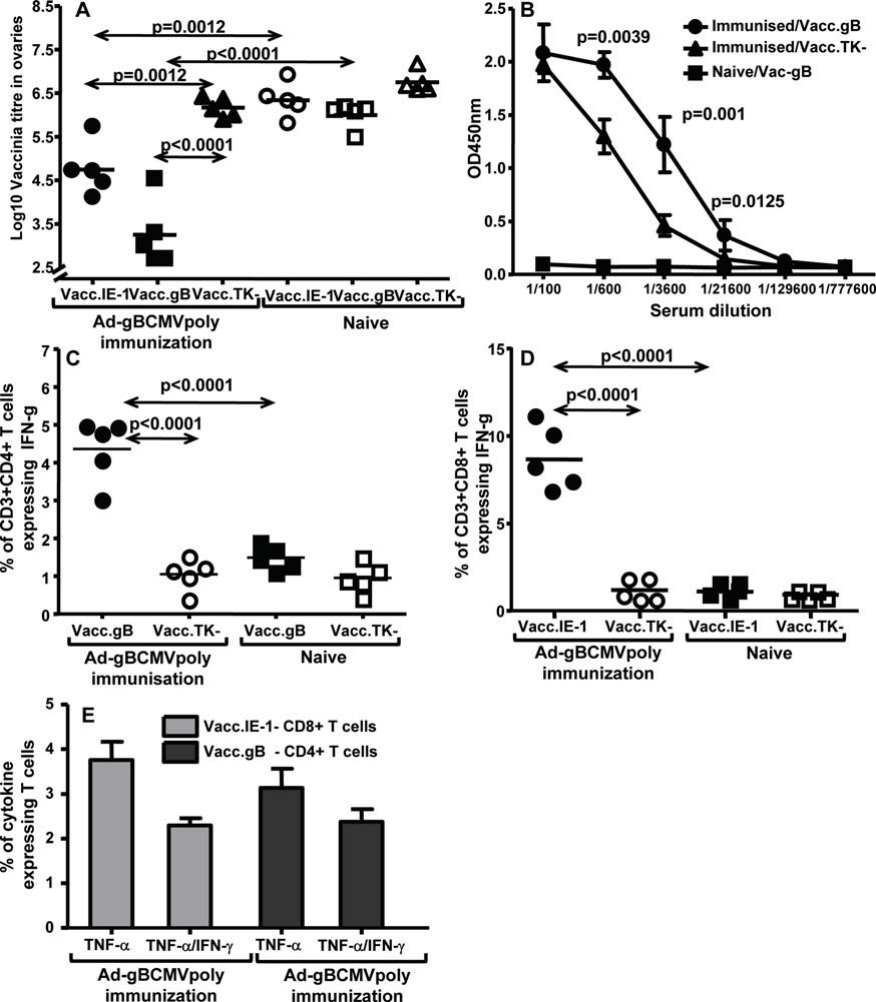
Ad-gBCMVpoly induced protection against challenge with recombinant vaccinia expressing gB or IE-1 protein. HHD-2 mice were immunised with Ad-gbCMVpoly vaccine and 21 days following vaccination these mice were challenged (intraperitoneal) with recombinant vaccinia encoding gB (Vacc.gB), IE1 protein (Vacc.IE-1) or control vaccinia (Vacc.TK^−^) at 10^7^ pfu virus/mouse. Ovaries, splenocytes and peripheral blood samples were collected four days later and used for assessing viral load, antigen-specific T cell response and gB-specific antibody response. *A,* Virus titres in the ovaries of Ad-gBCMVpoly immunised or naïve HHD-2 mice challenged with Vacc.IE-1, Vacc.gB or Vacc.TK^−^. *B,* gB-specific antibody response in Ad-gBCMVpoly immunised or naïve HHD-2 mice challenged with Vacc.gB or Vacc.TK^−^. *C,* *Ex vivo* gB-specific CD3^+^CD4^+^ T cell response in Ad-gBCMVpoly immunised or naïve HHD-2 mice challenged with Vacc.gB or Vacc.TK^−^. Splenocytes from these mice were stimulated with recombinant gB protein (40 µg/ml) overnight and then assessed for IFN-γ production using intracellular cytokine assay. *D,* *Ex vivo* IE-1-specific CD3^+^CD8^+^ T cell response in Ad-gBCMVpoly immunised or naïve HHD-2 mice challenged with Vacc.IE-1 or Vacc.TK^−^. Splenocytes from these mice were stimulated with the peptide epitope VLEETSVML (1 µg/ml) overnight and then assessed for IFN-γ production using intracellular cytokine assay. *E,* *Ex vivo* expression of IFN-γ and/or TNF-α by antigen-specific CD8^+^ and CD4^+^T-cells from Ad-gBCMVPpoly vaccinated mice, challenged with recombinant vaccinia encoding IE-1 or gB. Splenocytes from immunised mice stimulated with either gB protein or IE-1 peptide epitope overnight for intracellular cytokine assay. Data represent the percentage of TNF-α and IFN-γ & TNF-α expressing CD4^+^ or CD8^+^ T-cells. All statistical analyses were conducted using GraphPad Prism 4 software.

Having firmly established the immunogenicity of Ad-gBCMVpoly vaccine, the next set of experiments was designed to determine protective efficacy of this vaccine. Due to the species restriction, we challenge immunised HHD-2 mice with recombinant vaccinia encoding HCMV antigens (gB and IE-1) to evaluate the protective efficiency of the Ad-gBCMVpoly vaccine. Data presented in Figure 6A shows that HLA A2 mice immunised with Ad-gBCMVpoly vaccine showed significant reduction in the virus load following challenge with Vacc.gB and Vacc.IE-1. This reduction in the virus load was highly antigen-specific as the vaccinated or naïve animals challenged with Vacc.TK- or Vacc.gB, Vacc.IE-1 respectively showed minimal reduction in the viral load. Although Ad-gBCMVpoly immunized mice showed better protection against Vacc.gB when compared to Vacc.IE-1 (Figure 6A), this better protection was not due to anti-gB antibodies (Figure 6B) as Vacc.gB was not neutralized by serum from immunized animals (data not shown), but due to gB-specific CD4^+^ T cell responses (Figure 6C). Nevertheless, the anti-gB humoral response should play an important role in human as it induces HCMV neutralizing antibodies. As expected, the reduction in the Vacc.IE-1 virus load in Ad-gBCMVpoly immunised mice was co-incident with the induction of VLE-specific CD8^+^ T cell responses (Figure 6D). It is important to note that Ad-gBCMVpoly immunised mice challenged with Vacc.gB or Vacc.IE-1 showed significantly higher humoral and T cell responses respectively when compared to mice challenged with Vacc.TK-. We also assessed the level of TNF-α expression by IFN-γ expressing CD4^+^ and CD8^+^ T-cells using intracellular cytokine assays. Data presented in Figure 6E shows that following stimulation with gB protein or HCMV IE-1 epitope, a large proportion of CD4^+^ and CD8^+^ T cells from these mice showed strong co-expression of IFN-γ and TNF-α.

### Expansion of multiple antigen-specific human CD8^+^ and CD4^+^ T cells following stimulation with Ad-gBCMVpoly

**Figure 7:**
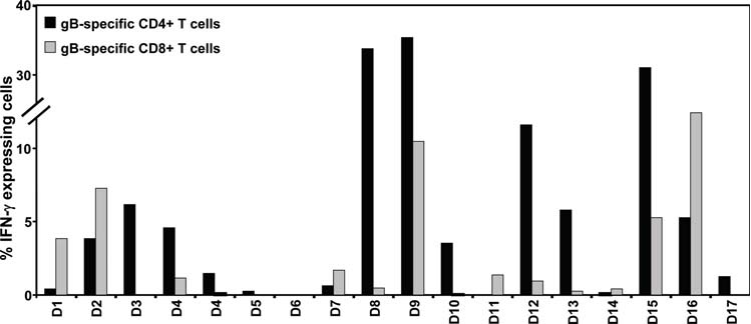
Expansion of gB-specific T cells following in vitro stimulation of human PBMC with Ad-gBCMVpoly. PBMC from a panel of healthy virus carriers (referred to as D1–D17) were co-cultured with autologous PBMC infected with Ad-gBCMVpoly (MOI: 5∶1 or 1∶1) at a responder to stimulator ratio of 2∶1. These cultures were supplemented with rIL-2 (10 U/ml) on day 3 and every 3–4 days thereafter. On day 14, these T cell cultures were tested against a panel of pooled overlapping gB peptides (20 aa long, overlapping by 10 aa) using intracellular cytokine assays. The data presented in the figure shows the percentage of gB-specific CD8+ and CD4+ T cell recovered from each donor following stimulation with Ad-gBCMVpoly.

Another important aspect of the current study was aimed at exploring the potential efficacy of Ad-gBCMVpoly to recall memory T cell responses from healthy seropositive individuals. PBMC from healthy donors were stimulated with irradiated autologous PBMC-infected with Ad-gBCMVpoly. Following stimulation these T cells were assessed for antigen specificity using intracellular cytokine assays. Data for the gB-specific CD4^+^ and CD8^+^ T cell responses are summarised in Figure 7, while the T cell responses towards the epitopes within the polyepitope sequence are presented in Tables 2 & 3. To identify the gB-specific T cell responses we used an overlapping set of peptides based on the gB sequence from Ad169 strain of HCMV. This analysis showed that following stimulation with Ad-gBCMVpoly, more than 88% of the individuals showed expansion of gB-specific CD4^+^ T cells. These T cell expansions raged from 2–36% of the total CD3^+^ CD4^+^ T cells (Figure 7). CD8^+^ T cell responses directed towards gB epitopes were detected in 70.5% donors which ranged from 2–15% of the total CD3^+^ CD8^+^ T cells. T cells from each donor recognized multiple gB epitopes and most of the donors demonstrated a selective expansion of gB-specific CD4^+^ or CD8^+^ T cells.

**Table 2:** CD8^+^ T cell responses in healthy virus carriers following stimulation with Ad-gBCMVpoly^a^.

HCMV Epitopes	D1 (A1 A29 B8 B44)	D2 (A1 A3 B7 B8)	D3 (A1 A24 B8 B14)	D4 (A23 A24 B27 B41)	D5 (A11 A24 B35 B60)	D6 (A1 A31 B8 B51)	D7 (A1 A11 B8 B35)	D8 (A24 A26 B15 B62)	D9 (A2 A11 B13 B27)	D10 (A2 B35 B57)	D11 (A3 A23 B35 B44)	D12 (A24 A26 B35 B38)	D13 (A1 A2 B7 B57)	D14 (A1 A2 B44)	D15 (A31 A33 B35 B58)	D16 (A1 A2 B7 B37 Cw6&7)	D17 (A1 A1 B8 B8 Cw7)	D18 (A2 A68 B8 B15 Cw4&7)
VTE (HLA A1; pp50)	+++^b^	++++	++++			++++	++++						+++	++++		++++	++	
KPG (HLA B35; pp65)					+/−		+/−			+++	+/−	+/−			++++			
NTD (HLA A1; gB)	+/−	+/−	+/−			+/−	+/−						+/−	+/−		+/−	+/−	
VLE (HLA A2; IE-1)									++++	++++			++++	++		+/−		++++
NLV (HLA A2; pp65)									++	++++			++++	+/−		+/−		++++
RIF (HLA A2 pp65)									+++	++++			+/−	+/−		+/−		+++
IIY (HLA A2 IE-2)									+/−	+++			+++	+/−		++		++
CVE (HLA A1; IE-1)	+/−	+/−	+/−			+/−	+/−						++	+/−		+/−	+/−	
VLA (HLA A2; IE-1)									+/−	++			+/−	+/−		+/−		+/−
AVG (HLA A2; gB)									+/−	+++			+/−	+/−		++		+/−
TVR (HLA A3; pp50)		+/−									+/−							
IMR (HLA A3; gB)		+/−									+/−							
GPI (HLA A11; pp65)					+/−		+/−		+/−									
AYA (HLA A23/A24; pp65)			+++	++	+/−			++			++++	+/−						
QYD (HLA A24; pp65)			+/−	+/−	+/−			++				++						
YVK (HLA A26; pp65)								++				++						
DIY (HLA A26; pp65)								+/−				+/−						
VFE (HLA A29; gB)	+/−																	
KARD (HLA B7; pp150)		+/−											+/−			+/−		
KARA (HLA B7/B8; IE-1)	+/−	+/−	+/−			+/−	+/−						+/−			+/−	+/−	+/−
TRA (HLA B57/B58; pp65)										++++			+/−		+/−			
HEL (HLA DR11; gH)				+/−														
DDY (HLA DR7; gB)										++								
QIK (HLA B8; IE-1)	++++	+++	++			++++	+++										++++	++++
RRR (HLA B8/B27; pp65)	+/−	+/−	+/−	+/−		+/−	+/−		+/−								+/−	+/−
ARV (HLA B27; pp28)				++++					+/−									
NVR (HLA B7; pp150)		+/−											++			+/−		
CPS (HLA B35; pp65)					+/−		++++			++++	++++	+/−			+/−			
QAR (HLA B7; pp65)		+/−											+/−			+/−		
ELR (HLA B8; IE-1)	+++	++	+++			++++	++++										++++	+++
IPS (HLA B35; pp65)					++++		+/−			+/−	++++	+/−			+++			
FEQ (HLA B41; IE-2)				++++														
YAY (HLA B41; gB)				++++														
QEF (HLA B44; pp65)	+/−			++++							+/−			+/−				
YEQ(HLA B44; pp50)	++										+/−			+/−				
QEP (HLA B44; pp65)	+/−									+++	+/−			+/−				
SHE (HLA B44; pp65)	++										++			+/−				
QAI (HLA B57/B58 pp65)															++++			
CED (HLA B40/60; pp65)					+/−													
KMQ (HLA B40/60; pp65)					+/−													
ATV (HLA A11; pp65)					+/−		+/−		+/−									
HER (HLA B40/60; pp65)					+/−													
DAL(HLA B51; pp65)						+/−												
VYA (HLA A24; pp65)			+/−	+/−	+/−			+++				++						
PTF (HLA B38; pp65)				+/−							+/−	+/−						
QMW (HLA B52; pp65)																		

a T cell response was assessed by intracellular cytokine assays for the secretion of IFN-γ.

b Proportion of IFN-γ-secreting CD8^+^ T cells +/−: <1%, ++: 1–4%, +++: 5–10%, ++++: >10%.

**Table 3:** CD4^+^ T cell responses in healthy virus carriers following stimulation with Ad-gBCMVpoly^a^.

HCMV Epitopes	D1 (A1 A29 B8 B44)	D2 (A1 A3 B7 B8)	D3 (A1 A24 B8 B14)	D4 (A23 A24 B27 B41)	D5 (A11 A24 B35 B60)	D6 (A1 A31 B8 B51)	D7 (A1 A11 B8 B35)	D8 (A24 A26 B15 B62)	D9 (A2 A11 B13 B27)	D10 (A2 B35 B57)	D11 (A3 A23 B35 B44)	D12 (A24 A26 B35 B38)	D13 (A1 A2 B7 B57)	D14 (A1 A2 B44)	D15 (A31 A33 B35 B58)	D16 (A1 A2 B7 B37 Cw6&7)	D17 (A1 A1 B8 B8 Cw7)	D18 (A2 A68 B8 B15 Cw4&7)
VTE (pp50)	++^b^	++	+++			+++	++						+/−	+++		++	++	
KPG (pp65)					++		+/−			++	+/−	+/−			++			
NTD (gB)	+/−	+/−	++			+/−	+/−						+/−	++		+/−	+/−	
VLE (IE-1)									+/−	+/−			+/−	++		+/−		+/−
NLV (pp65)									++	+/−			+/−	++		+/−		++
RIF (pp65)									+/−	+/−			+/−	++		+/−		+/−
IIY (IE-2)									+/−	+/−			+/−	+/−		+/−		+/−
CVE (IE-1)	+/−	+/−	++			+/−	+/−						+/−	++		+/−	+/−	
VLA (IE-1)									+/−	+/−			+/−	++		+/−		+/−
AVG (gB)									+/−	+/−			+/−	++		+/−		+/−
TVR (pp50)		+/−									+/−							
IMR (gB)		+/−									+/−							
GPI (pp65)					+/−		+/−		+/−									
AYA (pp65)			+/−	+/−	+/−			++			++	+/−						
QYD (pp65)			+/−	++	+/−			++				+/−						
YVK (pp65)								++				+/−						
DIY (pp65)								+/−				+/−						
VFE (gB)	+/−																	
KARD (pp150)		+/−											+/−			+/−		
KARA (IE-1)	+/−	+/−	++			+/−	+/−						+/−			+/−	+/−	+/−
TRA (pp65)										+/−			+/−		+/−			
HEL (gH)				+++														
DDY (gB)										++++								
QIK (IE-1)	+++	++	++			++	++										++	+/−
RRR (pp65)	+/−	+/−	+/−	+/−		+/−	+/−		+/−								+/−	+/−
ARV (pp28)				+/−					+/−									
NVR (pp150)		+/−											+/−			+/−		
CPS (pp65)					+/−		++			+/−	++	+/−			+/−			
QAR (pp65)		+/−											+/−			+/−		
ELR (IE-1)	++	+/−	++			+++	++										++	+/−
IPS (pp65)					+++		+/−			+/−	++	+/−			++			
FEQ (IE-2)				++														
YAY (gB)				++														
QEF (pp65)	+/−		++								++			++++				
YEQ(pp50)	+/−										++			++				
QEP (pp65)	+/−										+/−			++				
SHE (pp65)	++										+/−			+/−				
QAI (pp65)															+++			
CED (pp65)					+/−													
KMQ (pp65)					+/−													
ATV (pp65)					+/−		+/−		+/−									
HER (pp65)					+/−													
DAL(pp65)						+/−												
VYA (pp65)			++	+/−	+/−			++				+/−						
PTF (pp65)				++++							+/−	++++						
QMW (pp65)																		

a T cell response was assessed by intracellular cytokine assays for the secretion of IFN-γ.

b Proportion of IFN-γ-secreting CD4^+^ T cells +/−: <1%, ++: 1–4%, +++: 5–10%, ++++: >10%.

**Figure 8:**
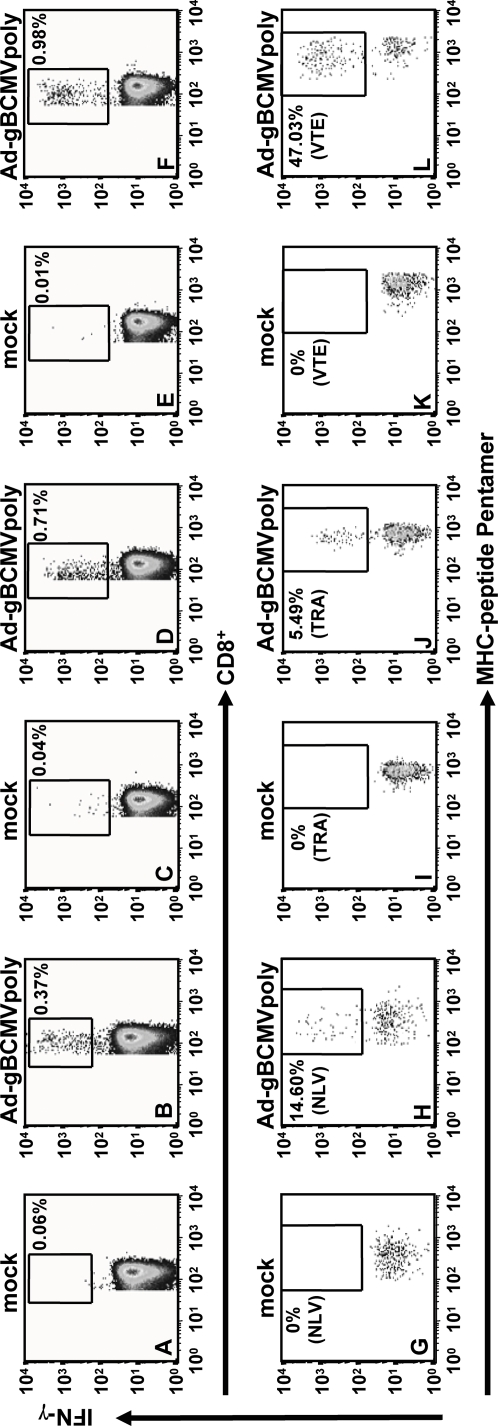
*Ex vivo* stimulation of human PBMC with Ad-gBCMVpoly. PBMC from healthy virus carriers were co-cultured with autologous PBMC infected with Ad-gBCMVpoly (MOI: 5∶1) at a responder to stimulator ratio of 2∶1 for 6 h. These T cells were then co-stained with anti-CD3, anti-CD8, PE-labelled anti-INF-γ antibody and APC-labelled MHC-peptide multimers. *A–F*, Percentage of CD8+ T cells expressing INF-γ following mock stimulation or Ad-gBCMVpoly stimulation. *G–L*, Percentage of MHC-peptide pentamer-positive cells expressing IFN-γ following mock stimulation or Ad-gBCMVpoly stimulation. Pentamers used for each of the HCMV epitopes are indicated in G–L.

Analysis of the T cell responses towards the epitopes within the polyepitope sequence revealed that there was a rapid expansion of CD8^+^ T cells following stimulation with Ad-gBCMVpoly which recognized multiple epitopes restricted through a number of HLA class I alleles (Table 2). In most cases, dominant CD8^+^ T cell expansions directed towards 2–3 different epitopes was observed; whilst in other donors (e.g. D9, D10 and D13) strong T cell reactivity towards more than five epitopes was observed. In vitro testing of these T cells also showed that these cells expressed high levels of CD107 and efficiently recognized HLA-matched HCMV-infected target cells (data not shown). These observations were also confirmed by ex vivo stimulating the PBMC from healthy virus carriers with Ad-gBCMVpoly. A representative data presented in Figure 8 clearly shows that ex vivo stimulation of PBMC rapidly stimulated HCMV epitope specific T cells and these cells showed strong expression of IFN-γ. Although the polyepitope sequence was predominantly based on CD8^+^ T cell epitopes, two previously mapped CD4^+^ T cell epitopes were also included in this sequence. As expected, a strong expansion of CD4^+^ T cells specific for these epitopes was observed, however unexpectedly, we also detected low to medium levels of expansion of CD4^+^ T cells which showed reactivity against HLA class I-restricted CD8^+^ T cell epitopes (Table 3). A careful analysis of these CD8^+^ T cell epitopes revealed that many of these sequences overlapped the CD4 epitopes mapped recently by other investigators [29], [30], [31].

## Discussion

The data presented in this study provides a highly efficient strategy for the prevention of HCMV disease in different clinical settings ranging from congenital infection to primary or reactivation of the virus in immunosuppressed adults. The importance of HCMV as the leading infectious cause of mental retardation and other abnormalities such as deafness in children has been emphasized by its categorization by the Institute of Medicine as a Level I vaccine candidate [i.e. most favourable impact–saves both money and quality-adjusted life years] [4]. Immunocompromised individuals such as transplant recipients and HIV-infected individuals with CD4 counts below 50/µl are also impacted by HCMV infection and this virus is regarded as the most important viral pathogen affecting transplantation, including both solid organ transplant and allogeneic hematopoietic stem cell transplant recipients [1], [32], [33]. Extensive studies over the last decade on the immunobiology of HCMV infection has provided detailed insight into the immune regulation of persistent HCMV infection in healthy virus carriers and individuals with HCMV-associated diseases [1]. Based on these observations, a number of attempts have been made to design a prophylactic vaccine for the control of HCMV infection. The first series of attempts focussed on the use of an attenuated form of the virus as a vaccine [6], [8], [34], [35] however, disappointing results coupled with the regulatory problems associated with the live attenuated HCMV vaccine prompted investigators to switch to the recombinant subunit approach [36], [37], [38], [39]. Although the subunit vaccine delivery systems and modalities based on HCMV encoded antigens such as gB, pp65 and IE-1 have failed to result in a licensed clinical product, interesting pre-clinical (based on animal models) and clinical data continues to accumulate demonstrating that subunit vaccination has a protective effect against congenital transmission [9], [10], [40], [41], [42].

It is now firmly established that long-term latent HCMV infection is very efficiently controlled by virus-specific CD4^+^ and CD8^+^ T cells [12], [13], [14], [15], [43]. Perturbation in the regulation of T cell control often triggers reactivation of HCMV and development of HCMV-associated diseases [17], [44], [45]. The concept that a vaccine based on T cell-mediated control would be effective in controlling HCMV diseases grew out of the pioneering work conducted by Riddell and colleagues, who showed that adoptive transfer of donor-derived virus-specific T cells alone were sufficient to reduce the incidence of HCMV disease in allogeneic hematopoietic stem cell transplant recipients [46], [47]. Over the last few years there has been a series of attempts to develop a highly tailored vaccine strategy designed to induce T cell immunity against pp65 and/or IE-1 antigens or defined T cell epitopes from these antigens [38], [48], [49].

While these strategies provided specificity and safety, their application at the population level are rather limited. Thus other approaches which target multiple antigens might be an advantage by providing wider coverage in different ethnic groups. Furthermore, inclusion of a virus neutralization component in the vaccine formulation has been argued by many investigators, especially in the context of congenital HCMV infection. Indeed the chimeric vaccine developed in this study induced high avidity humoral responses and cellular immunity with a single formulation and provided wider coverage through the inclusion of multiple T cell epitopes restricted through a range of HLA class I and II alleles. Our initial studies with a mixture of adenoviral vectors encoding HCMV polyepitope sequence and gB protein showed that it was possible to induce both humoral and cellular immune responses without compromising the immunogenicity of individual components of the vaccine. Taking into consideration these observations, we designed a chimeric vaccine in which the encoding sequence for the extracellular domain of gB was covalently linked with the polyepitope sequence. Extensive studies with this formulation provided further evidence that co-delivery of gB and the polyepitope as a single polypeptide was highly efficient in generating neutralizing antibodies responses and virus-specific CD8^+^ and CD4^+^ T cell responses in a murine model and healthy virus carriers. Subsequently, we employed an experimental animal model system to determine whether immunisation of HLA A2 transgenic mice with Ad-gBCMVpoly is capable of reducing infection with a recombinant vaccinia virus expressing HCMV antigens (i.e. gB and IE-1). These mice not only showed induction of a strong CD4^+^ and CD8^+^ T cell response following immunisation but also acquired strong resistance to virus infection. Interestingly, Ad-gBCMVpoly immunized showed better protection against Vacc. gB when compared to Vacc.IE-1, which suggested that gB-specific CD4^+^ T cell responses could also inhibit Vacc.gB virus.

Another important outcome of this study was the longevity of the immune responses induced by the chimeric HCMV vaccine which is particularly critical for the vaccine designed to control congenital infection/disease where long-term memory response over multiple years would be essential. Although the studies outlined here does not allow any firm conclusions on the efficacy of the chimeric polyepitope-based vaccine in humans, it does clearly show that a formulation based on gB and HCMV T cell epitopes can be used as immunogens to induce efficient humoral and T cell responses in vivo. It is important to stress here that a polyepitope-based vaccine for HCMV has a number of advantages over the traditionally proposed vaccines, which are based on either full-length HCMV antigens or synthetic peptide epitopes. There is now convincing evidence that polyepitope proteins are extremely unstable and are rapidly degraded by the proteasome dependent pathway as a result of their limited secondary and tertiary structure [18]. The rapid degradation of these polypeptides dramatically enhances endogenous presentation of peptide epitopes through the class I and II pathway. On the other hand the full-length HCMV protein antigens are unlikely to be degraded rapidly and may also initiate various intracellular signalling events leading to the interference of presentation of epitopes from other antigens [50], [51]. Finally, the polyepitope-based vaccine is likely to overcome any potential problem of reinfection with different strains of HCMV and unique HLA types in different ethnic groups of the world.

There is an emerging argument that HCMV vaccine efforts should focus on the development of formulation(s) which are designed to limit or prevent HCMV related diseases rather than to prevent infection itself [11]. This contention is supported by extensive studies in humans which revealed that although the immune responses generated during natural HCMV infection are unable to clear the latent virus, this response is sufficiently competent to keep the virus under control and restrict virus replication [13], [14], [15]. Furthermore, in immunocompromised patients such as HIV-infected individuals and transplant recipients, HCMV related pathogenesis is generally due to reactivation rather than primary infection. Considering the limited efficacy of the currently available HCMV vaccine formulations in protecting against infection in preclinical and clinical studies, we propose that a vaccine to limit or prevent HCMV related disease rather than infection itself is more realistic in the near future.

## Materials and Methods

### Construction of recombinant adenovirus encoding HCMV polyepitope, gB and gB-HCMV polyepitope fusion protein

The amino acid sequence of the 46 contiguous HLA class I and class II-restricted T cell epitopes (Table 1) were translated to the nucleotide sequence using human universal codon usage. Oligonucleotides (102–107mer long) overlapping by 20 base pairs and representing the polyepitope DNA sequence, were annealed together by using Splicing by Overlap Extension and stepwise asymmetric PCR [16]. The final PCR product was cloned into pBluescript II KS^+^ phagemid (Agilent Technologies, Melbourne, Australia) encoded a Kozak sequence, Start methionine followed by 46 contiguous HLA class I and class II-restricted epitopes. The HCMV sequence encoding glycoprotein B (gB) was amplified from the AD169 virus stock by PCR using gene specific primers. This PCR product was designed to encode gB sequence from the alanine residue at position 31 to valine at position 700 with the deletion of the signal sequence. Following amplification the DNA was cloned into pBluescript II KS^+^ phagemid and confirmed by DNA sequence analysis. For the expression of the gB-HCMV polyepitope fusion protein the recombinant HCMV polyepitope insert was excised from the pBluescript II KS^+^ phagemid and cloned into the gB pBluescript construct.

The assembly and production of the recombinant Ad5F35-based adenoviruses was completed in three stages using a highly efficient, ligation-based protocol of the Adeno-X System (CLONTECH, Palo Alto, CA) (See Figure 1). Firstly, inserts were excised from each of the constructs in pBluescript II KS^+^ phagemid using Xba I/Kpn I restriction enzymes and cloned into the pShuttle expression vector. Following amplification in E.coli, the expression cassette from pShuttle was excised using I-Ceu I/PI-Sec I homing enzymes and cloned into an Ad5F35 expression vector. The recombinant Ad5F35 vector was transfected into human embryonic kidney (HEK) 293 cells, and the recombinant adenoviruses (referred to as Ad-CMVpoly, Ad-gB and Ad-gBCMVpoly) were harvested from the transfected cells by successive freeze-thawing cycles.

### Synthesis of Peptides

Peptides, synthesized by the Merrifield solid phase method, were purchased from Chiron Mimotopes (Melbourne, Australia), dissolved in dimethyl sulphoxide, and diluted in serum-free RPMI 1640 medium for use in standard T cell assays. Purity of these peptides were tested by mass spectrometery and showed >90% purity

### Animals and immunisation

HLA A2 transgenic mice (referred to as HHD-2) [52], were maintained under conventional conditions the animal facility at the Queensland Institute of Medical Research. These mice are knocked out for β2 microglobulin and H-2D^b^ and transgenic for a chimeric HLA-A2.1 with the α3 domain derived from H-2D^b^ to allow interaction with murine CD8 and a covalently attached human β2 microglobulin. These mice were immunised with varying doses of plaque forming units (PFU) of recombinant viruses (Ad-CMVpoly, Ad-CMVgB and Ad-gBCMVpoly) and HCMV-specific humoral and cellular immune responses were evaluated at various time points. Protocols were approved by QIMR animal ethics committee.

### ELISpot assay

The ELISPOT assay was used to detect HLA A2-restricted HCMV epitope-specific T cells following stimulation with synthetic peptide(s) as described previously [13]. Briefly, 2×10^5^ responding cells were incubated in triplicate with each peptide epitope (1 µg/ml) for 18 to 20 hrs in 96- well Multiscreen HA filtration plates (MAHA S4150, Millipore, Bedford, MA) coated with anti-IFN-γ monoclonal antibody (Mabtech AB, Nacka, Sweden). After incubation, the plates were extensively washed with Phosphate buffered saline with 0.5% Tween 20 and incubated with a second biotinylated anti-IFN-γ mAb followed by the addition of streptavidin conjugated alkaline phosphatase. Cytokine producing cells were detected as purple spots after a 30-min reaction with 5-bromo-4-chloro-3-indolyl phosphate and nitro blue tetrazolium. Spots were counted automatically using image analysis software. T cell precursor frequencies for each peptide epitope were based on the total number of cells and the number of spot forming cells (SFC) per well (average of 3 wells). Epitope-specific spots were calculated after subtraction of the number of spots in control wells consisting of cells without added peptide (average of six wells).

### Intracellular Cytokine Staining

Splenocytes from immunised mice or T cells from human donors were incubated for overnight at 37°C with HCMV peptide epitopes (1 µg/ml), or stimulator cells either pre-coated with HCMV peptide epitopes (1 µg/ml) or infected with recombinant vaccinia virus encoding HCMV antigens, in growth medium. Brefeldin A (BD Pharmingen, San Diego, CA) was added during the last 5 hour-incubation. For CD107a staining, anti-CD107a antibody was added one hour before the adding of Brefeldin A. These cells were then washed and incubated with PerCP-conjugated anti-CD8, FITC conjugated anti-CD4 and Allophycocyanin-conjugated anti-CD3 at 4°C for 30 mins. Cells were washed, then fixed and permeabilised with cytofix/cytoperm (BD Pharmingen) at 4°C for 20 minutes. Cells were then washed in perm/wash (BD Pharmingen), incubated with anti-IFN-γ and anti-TNF-α mAbs (BD Pharmingen) at 4°C for 30 mins, washed again with perm/wash, resuspended in PBS and analysed on a FACS Canto.

### Expansion of HCMV specific T-cells from healthy donors using Ad-gBCMVpoly

A panel of 17 human volunteers were recruited for this study. Each volunteer was asked to sign the consent form as outlined in the institutional ethics guidelines. For the expansion of specific T-cells, peripheral blood mononuclear cells (PBMC) were co-cultured in multi-well tissue culture plates in growth medium with either PBMC (2,000 rad) infected with Ad-gBCMVpoly (MOI of 10∶1) at a responder to stimulator ratio of 2∶1. On day 3, and every 3–4 days thereafter, the cultures were supplemented with growth medium containing recombinant IL-2 (kindly donated by NIH AIDS Research & Reference Reagent Program). These T-cell cultures were assessed for HCMV epitope-specific reactivity on days 10–17.

### ELISA assay for anti-gB and anti-adenovirus antibody

Serum anti-gB or anti-adenovirus antibody titres were evaluated by ELISA as previously described [53]. Briefly, PVL microplate 96-well plates (MP Biomedicals, Sydney, Australia) pre-coated with recombinant HCMV gB protein or adenovirus were incubated with serially diluted serum samples for 2 hours at room temperature. After washing with PBS-Tween-20 (PBST), plates were incubated with HRP-conjugated sheep anti-mouse Ig antibody (murine samples) or HRP-conjugated sheep anti-human Ig antibody (human samples) for 1 hour. These plates were washed and incubated with 3.3′, 5.5′-tetramethylbenzidine substrate solution (PanBio, Brisbane, Australia) and the OD at 450 nm was analysed using an ELISA reader. The isotypes of anti-gB antibodies in serum samples were determined by ELISA as described above using the mouse monoclonal antibody isotyping reagent kit (Sigma, IS02-1 kit, Sydney, Australia) according to the manufacturer's protocol.

Antibody avidity was evaluated as previously described [54]. Briefly after incubation of plates with serum samples as described above, 5 M Urea (in PBST) was then added to half of the wells for dissociation and the other half received PBST without urea. After incubation for 30 min, plates were washed with PBST and incubated with HRP-conjugated sheep anti-mouse Ig antibody (murine samples) or HRP-conjugated sheep anti-human Ig antibody (human samples) for 1 hour and completed using the standard ELISA. The avidity indices were calculated as the ratio of the OD values with urea divided by the OD values without urea and expressed as a percentage.

### CMV microneutralization assay

The neutralizing activity of the anti-gB antibody response in vaccinated animals was assessed as described previously [23]. Briefly serum samples were initially incubated at 56°C for 30 minutes to inactivate complement, followed by serial dilution (25 µl/well) with DMEM medium in 96-well “U” bottom plates. In each well an equal volume of HCMV Ad169 was added and incubated at 37°C for 2 h. This virus was then transferred to infect monolayer of human fibroblast MRC-5 cell culture in 96 well flat bottom plates with 80–90% confluence. After 2 h, plates were washed with DMEM and 200 µl DMEM with 10% FCS were added to each well and then incubated at 37°C for 16–18 h. After incubation, cells were fixed in 100% methanol, incubated with peroxidase blocking reagent (Chemicon, S2001) and then reacted with mouse anti-CMV IE-1/IE-2 monoclonal antibody (Clone MAB810, Chemicon) followed by HRP-conjugated sheep anti-mouse Ig (Chemicon, AP326P). Finally cells were stained with DAB+ substrate (Chemicon, K3467) according to manufacturer's protocol. The numbers of nuclei with brown colour staining were counted using inverted microscope. The neutralizing titre was calculated as the reciprocal of sera dilution that gave 50% inhibition of IE-1/IE-2-expressing nuclei.

### Vaccinia virus recombinant

Recombinant vaccinia constructs encoding HCMV antigens IE-1 (Vacc.IE-1), gB (Vacc.gB) and a negative control vaccinia virus construct made by insertion of the pSC11 vector alone, which is negative for thymidine kinase (Vacc.TK^−^), have been previously described [13].

### Protection assay

HHD-2 mice were intramuscularly immunised with the indicated vaccine on day 0, followed by intraperitoneal challenge with recombinant vaccinia virus expressing different proteins from HCMV antigens (Vacc.IE1 or Vacc.gB) at a dose of 10^7^ pfu/mouse on day 21. Mice were then sacrificed 4 days later, spleens collected to evaluate epitope-specific T cell response by IFN-γ ICS assay, ovaries collected to determine vaccinia virus load by plaque assay on monkey fibroblast CV-1 cells, and sera collected to evaluate anti-gB Ab titres by ELISA. To determine vaccinia viral titres, monolayers of CV-1 cells in a 6 well flat bottom plates were incubated for 2 h at 37°C with serially diluted ovary lysates. After incubation, 2 ml of RPMI1640 medium supplemented with 2% FCS and 0.75% methylcellulose was added to each well and incubated for further 3 days. After three days, plates were washed with PBS and stained with crystal violet solution (Sigma, HT901) at a working concentration (0.1% crystal violet in 15% ethanol) for 30 min and the number of plaques were counted using standard procedures.
